# Biomarker recommendation for PD‐1/PD‐L1 immunotherapy development in pediatric cancer based on digital image analysis of PD‐L1 and immune cells

**DOI:** 10.1002/cjp2.152

**Published:** 2020-01-10

**Authors:** Manuel A Silva, Nicolas Triltsch, Simon Leis, Ivan Kanchev, Tze Heng Tan, Benjamine Van Peel, Marian Van Kerckhoven, Vanessa Deschoolmeester, Johannes Zimmermann

**Affiliations:** ^1^ Department of Translational Medicine, Clinical Biomarkers and Companion Diagnostics Merck KGaA Darmstadt Germany; ^2^ Department of Image Analysis Professional Services Definiens GmbH Munich Germany; ^3^ Department of Clinical Operations and Assay Development HistoGeneX Antwerp Belgium

**Keywords:** FoxP3, CD3, CD8, CD45RO, CD68, immunohistochemistry, digital pathology, tumor infiltrating lymphocytes

## Abstract

Anti‐PD‐1/PD‐L1 immunotherapy could offer an alternative to traditional chemo‐ and/or radiotherapy to treat pediatric cancer patients. To unveil the potential benefit of this new therapeutic approach, the prevalence of PD‐L1 and other relevant immune markers using quantitative digital image analysis (DIA) could help to clarify this point. A bridging study was first conducted using commercially available normal formalin‐fixed paraffin‐embedded (FFPE) tonsils to compare immunostaining patterns and intensities from PD‐L1, tumor infiltrating lymphocyte (TIL) markers CD3, CD8, FoxP3, CD45RO, and macrophage marker CD68 in adult (*n* = 5) and pediatric (*n* = 10) samples. Then, commercially available pediatric FFPE tumor samples from five prevalent pediatric solid tumor indications: ganglioneuroblastoma (*n* = 7); neuroblastoma (*n* = 23); nephroblastoma (*n* = 30); osteosarcoma (*n* = 24); and rhabdomyosarcoma (*n* = 25) were immunostained and their images (*n* = 654) digitally analyzed using predefined algorithms. The qualitative analysis of staining patterns and intensities in all 15 tonsils for all 6 biomarkers was similar regardless of age category. Quantitative DIA showed that PD‐L1 values varied across cancer‐types, nephroblastoma having the lowest counts. PD‐L1 counts in ganglioneuroblastoma, our pediatric indication with the highest average value, was approximately 12‐times lower than in a similar nonsmall cell lung cancer study, an indication approved for anti‐PD‐1/PD‐L1 immunotherapies. Variable values were measured for the TIL markers CD3, CD8, and CD45RO. FoxP3 was scant across all indications. The macrophage marker CD68 showed highest values in ganglioneuroblastoma, with lowest levels in nephroblastoma. In conclusion, the low PD‐L1 levels uncorrelated with TIL values from the present biomarker morphological study suggest that a PD‐L1 immunohistochemistry patient selection strategy used for anti‐PD‐1/PD‐L1 monotherapy in adult tumors may not succeed in these pediatric indications.

## Introduction

Pediatric oncology has seen great improvements in mortality and survival rates among children and adolescents due to the optimization of targeted combination chemotherapy, especially in hematological malignancies 1, 2. Despite the success, limited treatment approaches are available in solid neoplasia such as sarcomas and primary central nervous system cancer 1, 2, which is associated in part with acquired chemotherapy resistance 3, 4. Thus, novel treatment options like anti‐PD‐1/PD‐L1 checkpoint inhibitors could offer a therapeutic alternative to fulfill the current needs 5, 6. Such biologicals have been approved for the treatment of a variety of adult tumor types, unleashing antitumoral T‐cell adaptive immune responses suppressed by a PD‐1/PD‐L1 receptor‐ligand interaction between effector immune cells and tumor parenchyma 7, 8. Although no targeted PD‐1/PD‐L1 clinical trial has yet been completed, a phase I/II pediatric study performed in patients refractory to available treatments using the anti‐PD‐1 mAb Pembrolizumab showed a 8–9% overall response rate 9, 10, pointing out recent progress for checkpoint inhibitors in pediatric oncology.

A critical aspect to successfully develop anti‐PD‐1/PD‐L1 immunotherapies is the identification of individuals with a high probability of responding to treatment 6. In adults, this stratification is currently performed by selecting patients with elevated levels of PD‐L1 immunostaining in tumor areas by means of proprietary and/or developed companion diagnostic tests 11. Tumor tissue biomarker data from pediatric subjects is extremely limited and a low frequency of PD‐L1 positive tumors in children, except for Burkitt lymphoma and glioblastoma multiforme, has been reported in qualitative or semi‐quantitative assessments 9, 10, 12, 13, 14. More accurate evaluations using novel quantitative approaches, as reported in adult tumors 15, 16, are required to precisely identify individuals and cohorts with adequate levels of PD‐L1 immunostaining that allow effective development of anti‐PD‐1/PD‐L1 immunotherapies in the pediatric population. Additionally, a surrounding T‐cell rich microenvironment suppressed by the tumor is another feature required for eventual therapeutic success 11, 17, 18, 19. Then, analysis of different subpopulations of tumor infiltrating lymphocytes (TILs) by using biomarkers such as CD3 (pan T‐cell), CD8 (cytotoxic T‐cell), FoxP3 (regulatory T‐cell), and CD45RO (memory T‐cell) may help to identify predictive T‐cell signatures for immunotherapy. Some of these biomarkers like CD8 and FoxP3 are already considered prognostic factors in adult solid tumors as well as surrogate indicators for the strength of the host antitumoral immune response 20, 21, 22. Similarly, CD8/FoxP3 ratio and FoxP3 alone have been reported in pediatric studies to have prognostic value in high‐grade osteosarcoma and a subset of Hodgkin lymphoma 23, 24. In addition, another immune cell type with the potential to provide prognostic and predictive value is the tumor associated macrophage (TAM). These cells are CD68 positive and known to have functional plasticity ranging from anti‐ to pro‐tumoral effects depending on whether they represent M1 or M2 polarization, respectively 25. Although the role and significance of TAMs differ among adult solid cancer types 25, 26, elevated number of CD68 positive cells in pediatric osteosarcoma has been reported to have poor prognostic impact 14. All these prognostic biomarker data strongly suggest that not a single but rather a panel of biomarkers should be utilized to delineate potential predictive immune checkpoint inhibitor responses. In such a scenario, a technology like digital image analysis (DIA) has proven to accurately deliver multiple marker readouts when investigating broad adult tumor immune landscapes 15, 16, 27, 28.

Using commercially available tissues from children's prevalent solid‐tumors 29, 30, we performed the first pediatric DIA pathology study on immunohistochemistry (IHC) for PD‐L1 along with five other relevant immune cell biomarkers. The ultimate goal for this investigation was to unveil whether – or which of – these pediatric tumors might be potentially eligible for anti‐PD‐1/PD‐L1 checkpoint inhibition immunotherapy.

## Materials and methods

### Tissue, IHC, and scanning

A bridging study was first performed at HistoGeneX to confirm whether staining patterns and intensities for CD3, CD68, PD‐L1, CD8, FoxP3, and CD45RO in pediatric tissues are in line with what is observed in adult samples. Normal formalin‐fixed paraffin‐embedded (FFPE) tonsil tissue blocks from commercial sources were selected for this purpose after a tissue quality check, including 10 pediatric and 5 adult specimens (age‐range: 4–16 years and >18 years, respectively).

Validated HistoGeneX IHC laboratory developed tests (LDT) for diagnostics and established on Ventana Benchmark XT immunostainer (Roche Diagnostics, Mannheim, Germany) for CD3, CD8, CD45RO, and CD68; as well as on Ventana Benchmark ULTRA immunostainer (Roche Diagnostics) for PD‐L1; and in Lab Vision™ Autostainer (Thermo Fisher Scientific, Waltham, MA, USA) for FoxP3 were used (including their respective matching isotype controls) to stain four‐micrometer‐thick serial sections generated from the selected 15 FFPE tonsil specimens. A summary of all LDT IHC protocols and reagents is presented in Table 1. A subsequent qualitative manual IHC evaluation was conducted by two pathologists (MK and PV).

**Table 1:** Immunohistochemistry reagents and protocols

Primary Ab (clone)	Manufacturer (catalogue)	Ab isotype	Isotype control (catalogue)	Detection system (manufacturer; catalogue)	Counterstain (manufacturer; catalogue)	LDT number
CD3 (2GV6)	Ventana (790‐4341)	Rabbit IgG	Ventana (760‐1029)	UltraView DAB (Ventana; 760‐500)	Hematoxylin II (Ventana; 790‐2208)	TE‐IHC‐105
CD8 (C8/144B)	Dako (M7103)	Mouse IgG1, k	Dako (X0931)	UltraView DAB (Ventana; 760‐500)	Hematoxylin II (Ventana; 790‐2208)	TE‐IHC‐108
CD45RO (UCHL‐1)	Ventana (790‐2930)	Mouse IgG2a	Dako (X0943)	UltraView DAB (Ventana; 760‐500)	Hematoxylin II (Ventana; 790‐2208)	TE‐IHC‐51
CD68 (KP‐1)	Ventana (790‐2931)	Mouse IgG1, k	Ventana (760‐2014)	OptiView DAB (Ventana; 760‐700)	Hematoxylin II (Ventana; 790‐2208)	TE‐IHC‐107
FoxP3 (236A/E7)	eBioscience (14‐4777‐82)	Mouse IgG1	Abcam (Ab18448)	EnVision+ System‐HRP (Dako; K4002)	Hematoxylin (Dako; S3301)	TE‐IHC‐54
PD‐L1 (SP263)	Ventana (790‐4905)	Rabbit IgG	Ventana (790‐4795)	OptiView DAB (Ventana; 760‐700)	Hematoxylin II (Ventana; 790‐2208)	TE‐IHC‐87

In parallel, five solid‐tumor pediatric indications were selected based on prevalence according to European and USA epidemiology data 29, 30 (Table 2) in conjunction with availability of commercial FFPE human pediatric cancer specimens. Tumor tissue blocks from surgical resections collected before chemo/radiotherapy from patients with a diagnosis of ganglioneuroblastoma (*n* = 7); neuroblastoma (*n* = 23); nephroblastoma (*n* = 30); osteosarcoma (*n* = 24); and rhabdomyosarcoma (*n* = 25) were purchased from Cureline (Brisbane, CA, USA) as summarized in Table 3. As these specimens were commercially obtained, clinical data associated with each sample was limited and no therapeutic outcome information was available for any of these samples. Four‐micrometer‐thick serial sections per block were generated and stained for all six markers using the IHC protocols listed in Table 1. Slides were digitized using a 3dHistech Panoramic 250 FlashIII bright‐field scanner (3dHistech Ltd., Budapest, Hungary), and scanned at ×20. All whole‐slide digital images were assessed for scanning artifacts until images reached acceptable quality criteria.

**Table 2:** Prevalence of pediatric cancer in USA and Europe

	Pediatric cancer rate in the USA (cases/10^6^ person/year; age: 0–19) 29	Pediatric cancer rate in Europe (cases/10^6^ person/year; age: 0–14) 30	
		Western Europe	Eastern Europe
Leukemia	50	50.7	44.3
Brain and spinal cord tumors	49	38	30
Lymphoma	25	16.3	15.6
Neuroblastoma*/sympathetic nervous system†	8.3*	13†	12.5†
Nephroblastoma (Willms tumor)*/renal tumor†	6.3*	9.8†	9.8†
Osteosarcoma*/bone tumors†	5.4*	6.5†	6.0†
Rhabdomyosarcoma*/soft tissue sarcoma†	4.6*	9.7†	8.6†
Retinoblastoma	3.4	4.5	4.1

* Single indication.

† Tumor group.

**Table 3:** Tumor types analyzed by indication, site of origin, and patient treatment

Tumor name	Tumor site	No. of samples	Patient treatment
Ganglioneuroblastoma	Adrenal Gland	7	S + CTx
Neuroblastoma	Adrenal Gland	2	S + CTx
	Lymph Node	3	
	Pancreas	18	
Nephroblastoma	Kidney	30	S + CTx + R
Osteosarcoma	Femur	7	S + CTx
	Tibia	12	
	Radius	3	
	Fibula	2	
Rhabdomyosarcoma	Limb	17	S + CTx + R
	Back Muscle	3	
	Vulva	3	
	Vagina	1	
	Bladder	1	

Patient treatments: S, surgery; CTx, chemotherapy; R, radiotherapy. No information was provided on whether cases were refractory to chemotherapy, or on post‐treatment clinical outcome.

### Whole‐scan inspection, manual annotation, and DIA

**Figure 1:**
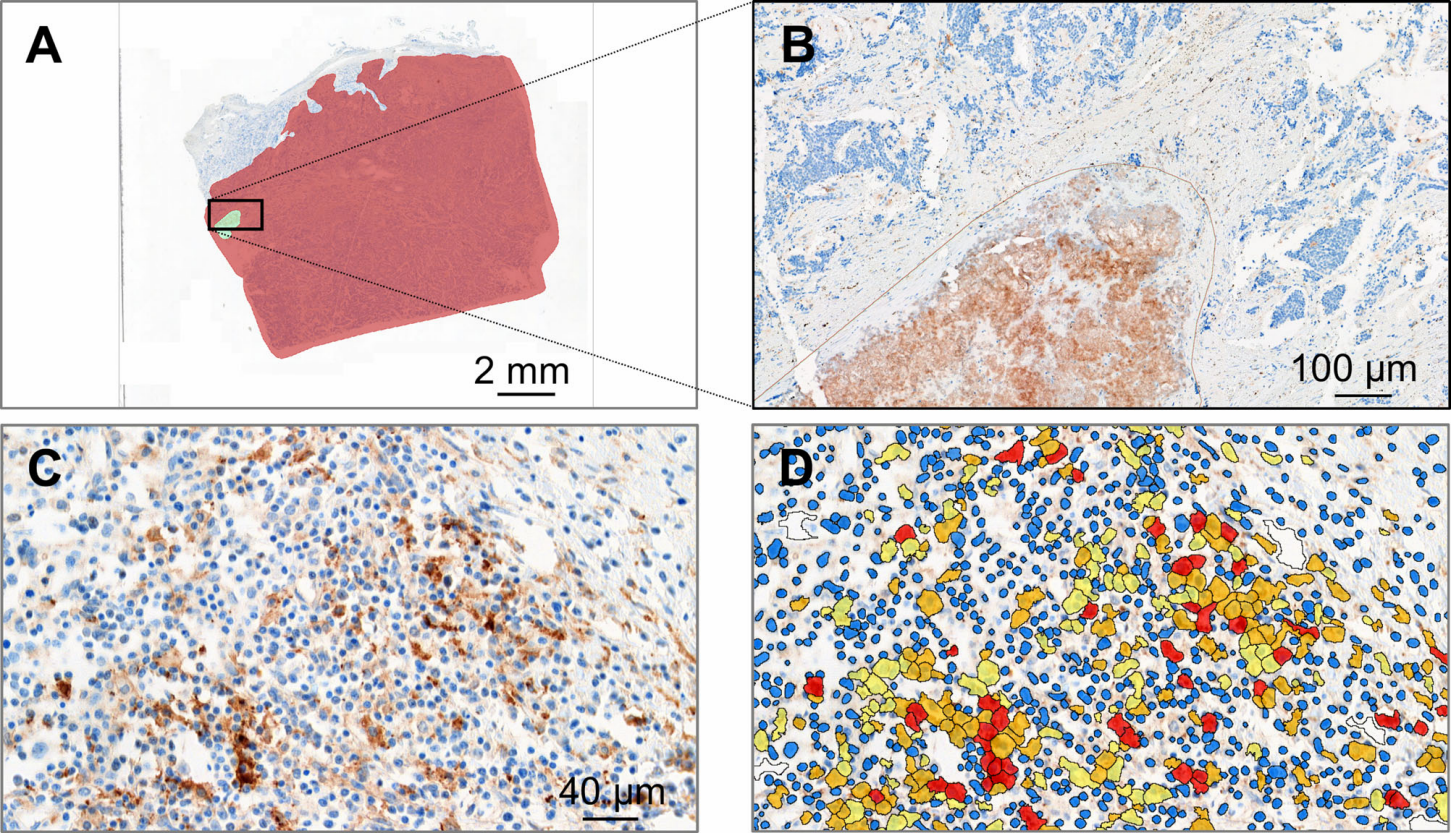
Example of the center of the tumor in red and exclusion annotations in green for a case of neuroblastoma in an overview (A) and at a closer view (B; location of the close‐up is indicated by the black rectangle in A). Original image of a PD‐L1 positive case of ganglioneuroblastoma (C) and corresponding DIA overlay (D). Colors indicate blue for negative nuclei, yellow, orange, and red for PD‐L1 positive cells of increasing staining intensities (low, medium, high, respectively).

Virtual slides were sent to Definiens (Munich, Germany), and a quality inspection for DIA executed in all native virtual slides (*n* = 654). Serial virtual slides were co‐registered using Definiens VeriTrova 2.2.1 software (Definiens) to ensure consistent generation of pathologist's annotations. Manual annotations to designate the center of the tumor (CT) as a region of interest (ROI) according to Definiens' standardized annotation guidelines were performed by a pathologist (IK) using Definiens VeriTrova. Region annotations of CT did not incorporate the invasive margin of the tumor mass and adjacent surrounding normal tissue. Artifacts such as folds and tears, mounting media artifacts, stain smudges, areas of necrosis, and cellular debris were addressed by manual exclusion annotations (Figure 1A,B).

Subsequently, virtual slides were submitted to DIA using Cognition Network Technology 31. Upon import into Definiens Developer XD 2.7.0 software (Definiens), a heuristic approach was pursued to accurately detect biological target structures 32 and specific biomarker algorithms previously developed (unpublished data) were applied for the analysis of these 5 pediatric indications (Figure 1C,D example for PD‐L1). Each algorithm determined cell positivity based on specific automated spectral measurements per cell developed for each biomarker considering staining saturation weighted by the relative area of stain within an individual cell. To this end, the DAB signal was transformed into a numerical scale ranging from 0 to 255. Spectral cut‐offs combined with size thresholds determined whether a stained object was or not considered a positive cell. False positive signals in IHC images, like anthracotic pigment and siderophages, were automatically subtracted by specific algorithms, taking into account color, size, and texture of such false positive structures.

PD‐L1 was the only biomarker analyzed considering staining intensity grades (low: spectral cut‐off range 5–25; medium: 25–65; high: above 65; and negative: below 5). These low, medium and high intensity grades were translated by our pathologists as +1, +2, and +3 staining intensities, similar to a previous nonsmall cell lung cancer (NSCLC) DIA study 15. For the other markers, signals above their respective spectral cut‐off (5 for CD3; 7 for CD45RO; 8 for CD8 and CD68; and 25 for FoxP3) were considered positive. Reliable detection of true positive FoxP3 staining could not be achieved in some cases of neuroblastoma and osteosarcoma due to recognition of pigmented dots so some virtual slides from these indications were not analyzed. A detailed segregation of the stromal tumor microenvironment (TME) area from the neoplastic cells could not be performed due to the broad morphological heterogeneity of the embryonal and mesenchymal compartments as well as unclear definitions of TME and neoplastic cells in pediatric tumor indications 33, 34, 35. DIA results were reported as the density of biomarker positive cells per square millimeter per ROI, and the percentage of biomarker positive cells per all analyzed cells per ROI, which include positive and negative immune as well as tumor cells.

### Statistical analysis

The distribution of the expression of the biomarkers across indications was plotted and pair‐wise comparisons made. Values are presented as median ± median absolute deviation (MAD). Bonferroni‐corrected Mann–Whitney *U* test *P* values for pair‐wise marker densities were generated and significant differences were reached with *p* < 0.05. The pair‐wise correlations of PD‐L1 level with the other five immune markers were plotted, and noncorrected Spearman's correlation coefficients (SCC) including corresponding *P* values generated. We only considered a correlation significant when *p* < 0.01 to provide a more conservative estimate due to multiple testing. Clustered heatmaps were computed to identify subgroups within each indication. A standardized *z*‐score for each individual marker was calculated across all indications, and data was clustered based on the Euclidian distance between the standardized *z*‐score and the marker level of each subject. Hot tumors were defined as cases with positive standardized *z*‐score. All analyses were performed using the open source programming language R (3.5.3); R Foundation for Statistical Computing, Vienna, Austria.

## Results

### Bridging study

**Figure 2:**
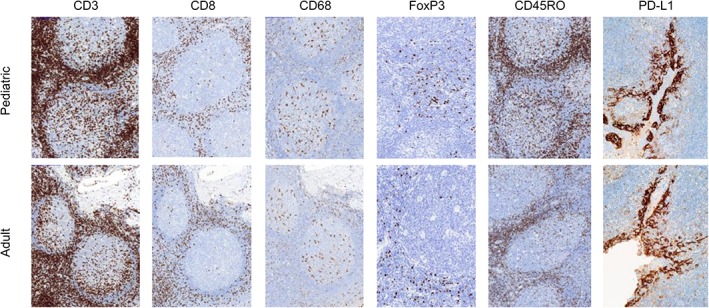
Representative micrographs of CD3, CD8, CD45RO, CD68 IHC (×10) as well as FoxP3 and PD‐L1 IHC (×20) in pediatric and adult tonsils, respectively, from the bridging study.

Qualitative manual analysis of staining patterns and intensities showed similar CD3, CD68, PD‐L1, CD8, FoxP3, and CD45RO immunophenotypes in all 15 tonsils regardless of age‐group category (Figure 2).

### DIA and quantification

**Figure 3:**
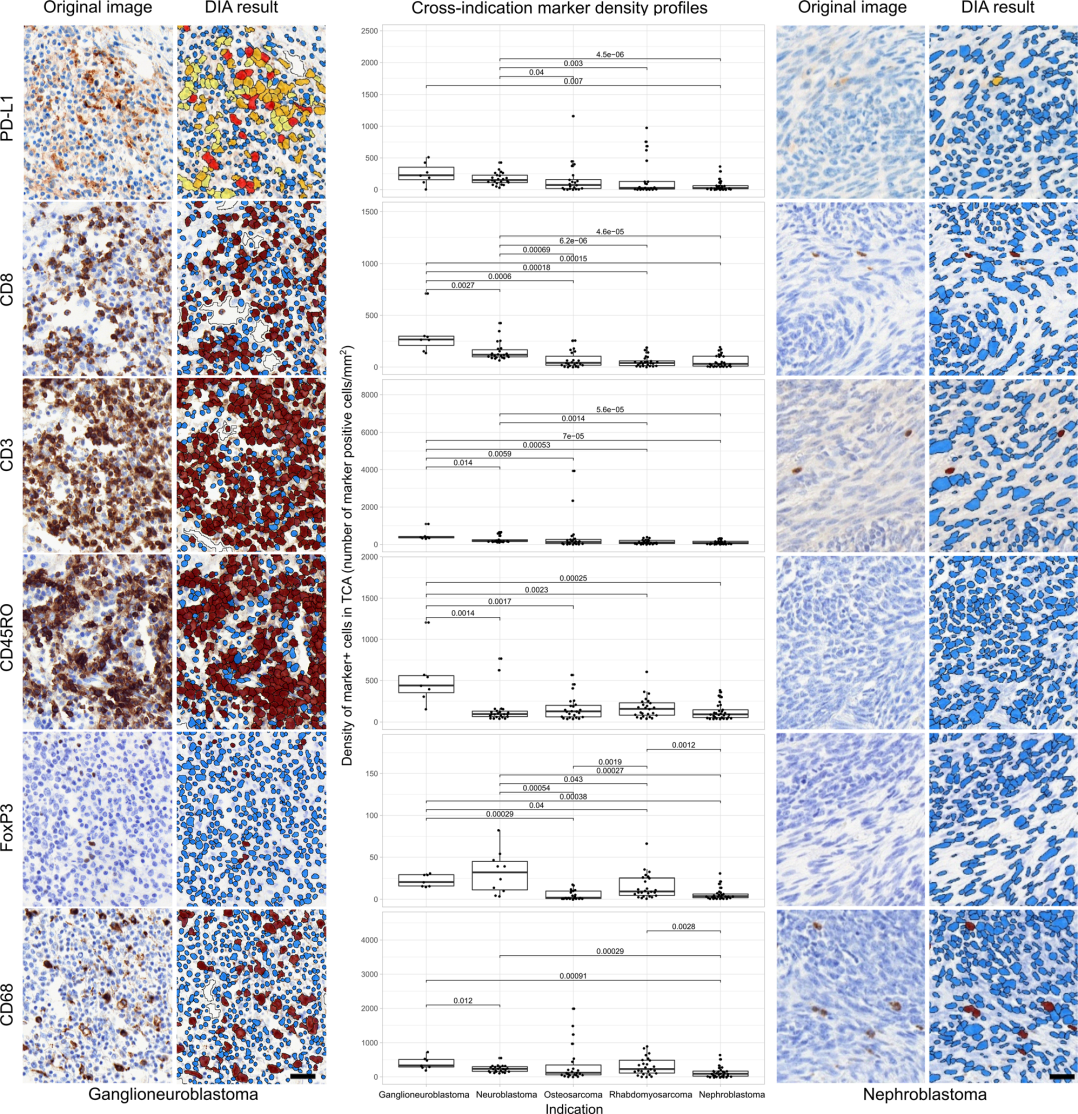
DIA results from IHC. Representative IHC stained images and their corresponding DIA segmentation overlays of CD3, CD8, CD45RO, CD68, FoxP3, and PD‐L1 IHC (×20) from ganglioneuroblastoma, the indication showing the highest PD‐L1 expression, far left, and from nephroblastoma, the indication showing the lowest PD‐L1 expression, far right. Same cases and similar regions from serial sections were selected. Classification colors indicate blue for negative nuclei, brown for CD3, CD8, CD45RO, CD68, and FoxP3 positive cells; and yellow, orange, and red for PD‐L1 positive cells of increasing stain intensity. The boxplots in the middle panel show distribution profiles of investigated biomarkers across investigated indications. Indications are sorted by descending order of mean PD‐L1 positive cell densities. Horizontal lines mark indication pairs where the differences in distribution are statistically significant and corresponding adjusted *P* values are indicated above.

Scans from all stained slides (*n* = 654) showed specific labeling for these 6 biomarkers, which were successfully processed except for 12 neuroblastoma and 2 osteosarcoma FoxP3 cases that were excluded from the analysis due to pigments interfering with the target stain chromogen. Densities of the investigated immune biomarkers across all tumor indications, expressed as number of positive cells per square millimeter, are presented in Figure 3 along with corresponding DIA results for selected indications with highest and lowest values for PD‐L1 positivity, ganglioneuroblastoma and nephroblastoma respectively. Percentages of positive cells per all analyzed cells can be found in supplementary material, Figure S1. A summary panel displaying representative IHC images and corresponding DIA per indication for each marker is presented in supplementary material, Figure S2 to show examples of the detection performance of our digital algorithms. Additionally, median values for all quantifications expressed by number of cells per square millimeter as well as percentages of positive cells per all analyzed cells are summarized in Table 4.

**Table 4:** Biomarker quantification in the center of the tumor

Biomarker	Indication	Number of positive cells per mm^2^ (median ± MAD)	% of positive cells per all analyzed cells (median ± MAD)
PD‐L1	A. Ganglioneuroblastoma	226 ± 157	2.8 ± 1.2
	B. Neuroblastoma	151 ± 99	1.8 ± 0.8
	C. Osteosarcoma	73 ± 96	1.7 ± 2.5
	D. Rhabdomyosarcoma	27 ± 34	0.4 ± 0.5
	E. Nephroblastoma	25 ± 31	0.3 ± 0.3
CD3	A. Ganglioneuroblastoma	398 ± 70	6.6 ± 2.4
	B. Neuroblastoma	181 ± 52	1.8 ± 0.6
	C. Osteosarcoma	128 ± 147	3.7 ± 4.2
	D. Rhabdomyosarcoma	106 ± 110	2.0 ± 1.9
	E. Nephroblastoma	79 ± 70	0.9 ± 0.9
CD8	A. Ganglioneuroblastoma	267 ± 53	3.4 ± 1.3
	B. Neuroblastoma	119 ± 36	1.4 ± 0.6
	C. Osteosarcoma	41 ± 42	1.0 ± 1.3
	D. Rhabdomyosarcoma	40 ± 39	0.7 ± 0.6
	E. Nephroblastoma	31 ± 34	0.3 ± 0.4
CD45RO	A. Ganglioneuroblastoma	308 ± 150	4.3 ± 1.5
	B. Neuroblastoma	43 ± 37	0.5 ± 0.4
	C. Osteosarcoma	68 ± 79	1.7 ± 2.3
	D. Rhabdomyosarcoma	91 ± 86	1.3 ± 1.6
	E. Nephroblastoma	41 ± 50	0.5 ± 0.6
FoxP3	A. Ganglioneuroblastoma	22 ± 9	0.4 ± 0.1
	B. Neuroblastoma	34 ± 31	0.4 ± 0.3
	C. Osteosarcoma	2 ± 2	0.04 ± 0.05
	D. Rhabdomyosarcoma	10 ± 9	0.2 ± 0.1
	E. Nephroblastoma	4 ± 3	0.04 ± 0.04
CD68	A. Ganglioneuroblastoma	376 ± 222	5.1 ± 2.0
	B. Neuroblastoma	269 ± 37	3.0 ± 0.4
	C. Osteosarcoma	146 ± 79	3.9 ± 2.3
	D. Rhabdomyosarcoma	263 ± 86	4.5 ± 1.6
	E. Nephroblastoma	114 ± 50	1.2 ± 0.6

MAD = median absolute value.

Prevalence of PD‐L1 positive cells in the CT varied across indications (Figure 3 and Table 4). When these indications were organized according to high‐to‐low PD‐L1 relative ranking based on their quantifications, the relative rankings based on densities matched the ranking based on percentages. Furthermore, the highest median value for PD‐L1 was observed in ganglioneuroblastoma followed by neuroblastoma and osteosarcoma. Notably, nephroblastoma showed the lowest PD‐L1 median value among all analyzed indications regardless of the parameter used. Also, of note is the relatively narrow range of the expression level of PD‐L1 among the ganglioneuroblastoma and neuroblastoma patients. Significant differences across indications are presented in Figure 3.

TIL marker prevalence also varied across indications (Figure 3 and Table 4). As in PD‐L1, the indication‐related ranking for CD3 and CD8 median values was the same for both densities and percentages. Moreover, ganglioneuroblastoma showed the highest median value for both markers (Table 4), reaching *p* < 0.05 when comparing densities versus all other indications as presented in Figure 3. The following indications showing higher CD3 and CD8 median values were neuroblastoma and osteosarcoma, respectively (Table 4). Nephroblastoma showed the lowest levels of both markers in the ranking. Additional significant differences between indications in CD3 and CD8 densities are shown in Figure 3. The same narrow distribution for the expression levels of CD3 and CD8 in ganglioneuroblastoma and neuroblastoma is also observed here.

In CD45RO, the relative rankings were different when median values were organized by densities or percentages for osteosarcoma and rhabdomyosarcoma (Table 4). However, the relative ranking was the same for the remaining indications, ganglioneuroblastoma having the highest CD45RO median values and nephroblastoma the lowest values, respectively (Table 4). In addition, ganglioneuroblastoma showed significant differences in CD45RO cell density versus all other indications (Figure 3).

The TIL marker FoxP3 showed the lowest counts when compared with all five other markers. Similar FoxP3 indication relative rankings were obtained when comparing median values expressed as densities or percentages (Table 4). Neuroblastoma and ganglioneuroblastoma scored the highest FoxP3 median values, whereas neproblastoma and osteosarcoma showed the lowest levels, respectively (Table 4). Significant differences in FoxP3 densities among indications are presented in Figure 3.

The indication relative ranking for CD68 showed discrepancies when values were expressed as densities or percentages (Table 4). However, CD68 median values were highest in ganglioneuroblastoma and lowest in nephroblastoma when expressed by either method (Table 4). Significant differences among CD68 densities between indications are shown in Figure 3.

### Correlation plots and heatmaps

**Figure 4:**
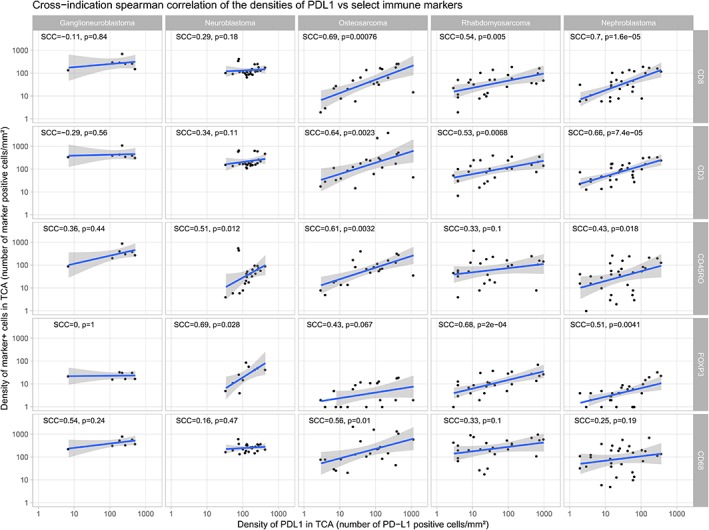
Correlation of PD‐L1 positive cell densities and other immune markers in the centers of the tumors. The Spearman correlation coefficients (SCCs) and their corresponding uncorrected *P* values are given by SCC and *p*, respectively.

When reviewing our density dataset for pair‐wise correlations (Figure 4), we noticed a poor association in ganglioneuroblastoma and neuroblastoma between PD‐L1 and most of the other immune markers. However, in osteosarcoma, rhabdomyosarcoma and nephroblastoma, there was a significant density correlation between PD‐L1 levels and the counts of the T‐cell markers CD3, CD8, and FoxP3, and to a lesser extent with CD45RO. The only significant density correlation between PD‐L1 and the macrophage marker CD68 was observed in osteosarcoma. Equivalent data distribution patterns but with different *P* values were detected when performing same analysis with the percentage dataset (see supplementary material, Figure S3).

**Figure 5:**
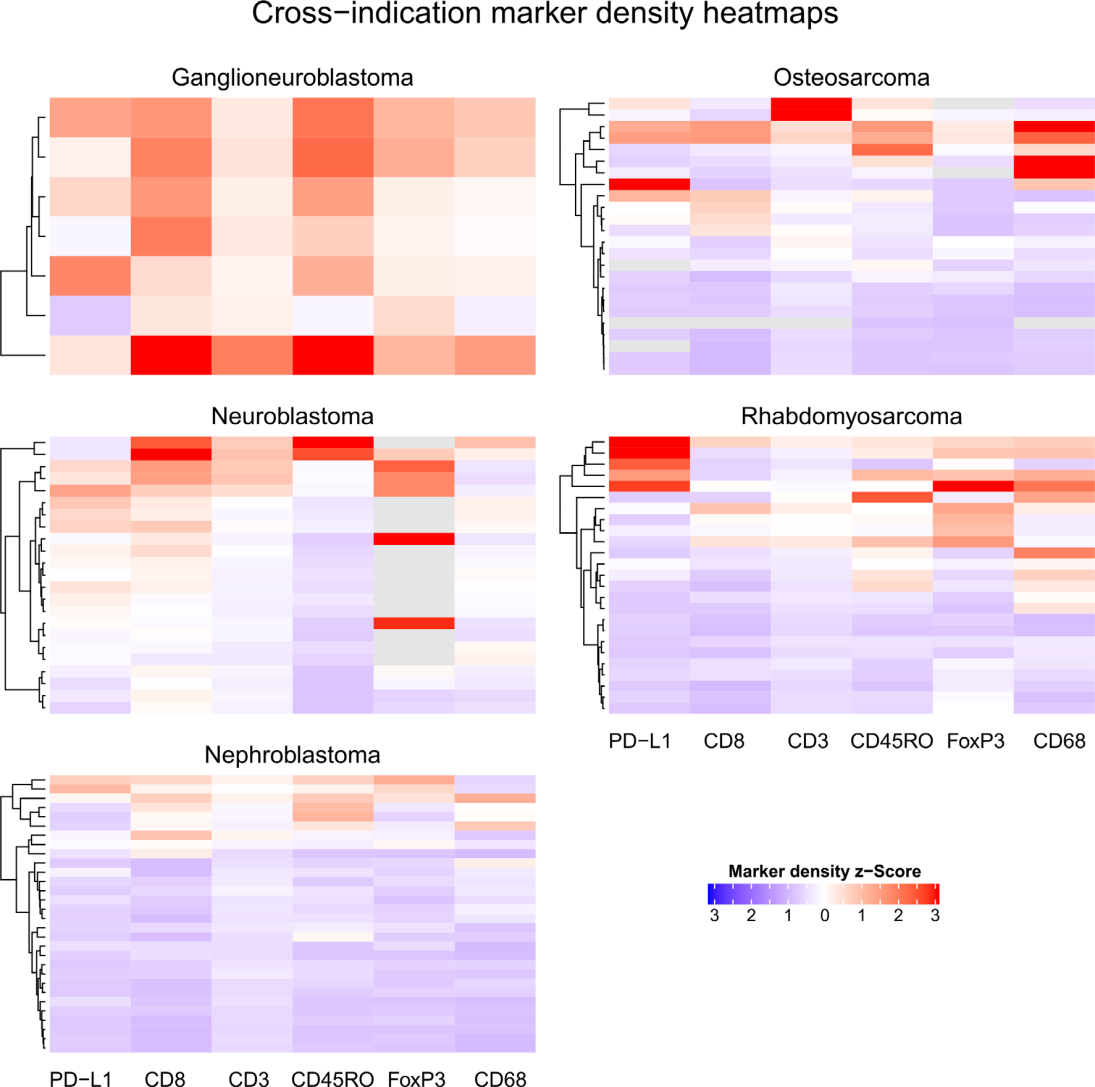
Clustered heatmaps of the individual cases (*y*‐axis) based on standardized expression levels of the individual markers across investigated indications. Red or blue indicates higher or lower than average levels, respectively.

Furthermore, we dissected the individual subjects within each indication and grouped them based on their relative density levels of the biomarkers studied (Figure 5). Of note is the presence of ‘hot’ tumors in ganglioneuroblastoma as all subjects had a higher than average expression of most markers when compared to all other indications. Among this small group of cases, the subject with highest TIL and CD68 marker density *z*‐scores had only a mid‐range PD‐L1 *z*‐score. The pattern for the other four indications was mixed, showing a subgroup of about 20% (21 cases) of relatively ‘hot’ tumors while the rest were relatively ‘cold’. It is worth mentioning that, in neuroblastoma, the two cases with strongest CD8 and CD45RO density *z*‐score presented mid/low range PD‐L1 *z*‐scores; while the two cases with highest FoxP3 density *z*‐scores shown low PD‐L1 *z*‐scores. In rhabdomyosarcoma, the cluster of five cases with highest PD‐L1 density *z*‐scores presented mid/low range density *z*‐scores for CD3, CD8, and CD45RO TIL markers. Moreover, only one of these cases presented a high FoxP3 density *z*‐score. In osteosarcoma, the case with the highest PD‐L1 density *z*‐score had low TIL *z*‐scores, while the two cases with highest CD3 *z*‐scores showed only mid‐range PD‐L1 density *z*‐scores. In addition, osteosarcoma presented the four cases with highest CD68 density *z*‐scores observed across all indications, two of these cases having high PD‐L1 and the other two low‐range PD‐L1 density *z*‐scores. In contrast to all other indications, nephroblastoma showed the three cases with highest PD‐L1 density *z*‐scores with similar TIL density *z*‐scores range, while the 21 cases with lowest PD‐L1 density *z*‐scores correlated with lowest TIL and CD68 density *z*‐scores. Similar results were observed with the percentage dataset (see supplementary material, Figure S4).

## Discussion

We have investigated immune biomarkers in whole tissue sections stained with validated IHC protocols and quantified them with a DIA platform. Evaluated were 109 pediatric surgical samples diagnosed with ganglioneuroblastoma, neuroblastoma, nephroblastoma, osteosarcoma, and rhabdomyosarcoma, which were collected before chemo/radiotherapy treatment. PD‐L1 IHC was performed in our study using a diagnostic assay with clone SP263, showing levels of PD‐L1 that varied across indications. The highest PD‐L1 values were detected in ganglioneuroblastoma followed by neuroblastoma and osteosarcoma, while the lowest values were observed in nephroblastoma. Variable levels were noted for the TIL markers CD3, CD8, and CD45RO; whereas FoxP3 was low across all indications. The macrophage marker CD68 exhibited the highest median value in ganglioneuroblastoma and lowest in nephroblastoma, respectively. Within each indication, except nephroblastoma, cases with the highest levels of PD‐L1 did not correlate with highest TIL marker levels (Type III paradigm; 17), suggesting that PD‐L1 and TIL markers may not follow the same immunobiology in these indications. In addition, percentages of PD‐L1 and FoxP3 positive cells per all analyzed cells in ganglioneuroblastoma (the investigated indication with the highest median values) were respectively equivalent only to 8.5 and 9.1% of the median of PD‐L1 and FoxP3 reported in similar NSCLC DIA 15; an indication approved for anti‐PD‐1/PD‐L1 immunotherapies, and the only one with published digital pathology data to allow us to put our pediatric results in perspective. Therefore, these results suggest that, from a phenotypic point of view, the low levels of PD‐L1 and immune markers shown in these pediatric solid tumors may not necessary support the PD‐L1 IHC biomarker approach used to develop anti‐PD‐1/PD‐L1 monotherapies in adult tumor indications. Furthermore, we speculate why anti‐PD‐1/PD‐L1 monotherapy in these pediatric diseases may not be effective.

In the present study, PD‐L1 positive cells were detected only in CT as a separation between neoplastic cells and TME was not possible due to high morphological tissue heterogeneity and unclear definition of those compartments in some of these pediatric indications 33, 34, 35. This issue restricted us from developing additional TME subcompartmental classification like the staining‐pattern method recently published in NSCLC 15. Our biomarker analysis method of reporting staining results as densities (number of positive cells per mm^2^) and percentages (number of positive cells per all analyzed cells) provided same relative rankings across indications, except for some discrepancies in CD45RO and CD68. PD‐L1 data from clinical trials have been systematically reported using variable cut‐off points based on amount of tumor‐cell membrane staining 11, 36, which restrained us from a direct comparison of our PD‐L1 results with the data presented by the pediatric KEYNOTE‐051 phase I/II study 9, 10. In contrast, our PD‐L1 dataset is comparable to a similar DIA performed in NSCLC where PD‐L1 results were also reported as positive cells per all analyzed cells in CT 15. In this adult study, the percentage of PD‐L1 positive cells was 32.9%, higher than the 2.8% found in the ganglioneuroblastoma group; the indication with the highest amount of PD‐L1 in the present study. Although this adult NSCLC study was performed using PD‐L1 clone 73‐10 and not SP263, this difference should not limit a comparison between both studies because both clones have similar analytical performance with equivalent specificity, with clone 73‐10 being somewhat more sensitive 37. Different scanners, software and IHC protocol variations may contribute to explaining the dissimilar results between both DIA studies. However, head‐to‐head comparisons between the DIA platforms used in these studies analyzing multiple single‐plex IHC biomarkers (including PD‐L1 and FoxP3), showed comparable outcome despite usage of different scanning and software (see supplementary material, Table S1). Therefore, the dramatic differences between our pediatric results and the ones published in NSCLC 15 are unlikely to be explained by these changes.

Of note, our PD‐L1 IHC quantification did not yield cases with zero values as we detected positive cells in all analyzed tissue samples, verified in a later quality assessment (data not shown). This is different from other pediatric IHC studies where manual scoring methods based on cut‐off points assigned zero values to single cases and/or groups 9, 10, 12, 13. This contrast highlights the higher sensitivity of our method to accurately evaluate tumor samples by applying continuous readouts instead of scoring schemes optimized for visual assessment. Despite this methodological divergence, our PD‐L1 counts are aligned with the ones reported in the aforementioned pediatric IHC studies as those also showed low prevalence of PD‐L1 in similar solid pediatric tumor indications 9, 10, 12, 13, 14. Again, a direct comparison of our results was not feasible because of the manual scoring methods applied in these other studies 9, 10, 12, 13. In addition, full analytical validation has not been performed for PD‐L1 clones such as E1L3N 12.

From a quantitative point of view, we hypothesized that the low amounts of PD‐L1 detected in our five pediatric cancer indications may not be sufficient for the development of effective anti‐PD‐1/PD‐L1 monotherapies, considering not only their lower levels compared with NSCLC (an indication for which immunotherapies are approved), but also because none of the patients diagnosed with these five solid tumor indications treated in the pediatric KEYNOTE‐051 phase I/II study 9, 10 responded to therapy, which includes individuals diagnosed with neuroblastoma (*n* = 9), nephroblastoma (*n* = 3), and osteosarcoma (*n* = 9). However, our samples were collected from patients undergoing standard treatment while patients enrolled in the pediatric KEYNOTE‐051 study were cases already refractory to standard therapies, suggesting that both groups of patients may not be comparable due to potential dissimilarities in disease stage and tumor immunophenotype 9, 10. We are also aware that a certain number of adult patients receiving anti‐PD‐1/PL‐L1 immunotherapy have responded to treatment even having tested negative for PD‐L1 IHC 11, 36, which could challenge our hypothesis. However, among other explanations, tissue specimens screened during clinical trial analysis may be small and not sufficient to generate an accurate representation of the immunophenotype of a whole tumor; especially relevant for heterogeneous markers like PD‐L1 11, 15, 36. For that reason, our study was conducted using surgical resections to assure that our results will provide a more representative assessment of each investigated tumor‐type. When considering other pediatric cancer indications, it is worth mentioning that one study showed high frequency of PD‐L1 positive tumors in pediatric glioblastoma multiforme and Burkitt lymphoma 13. Also, responders from the pediatric KEYNOTE‐051 phase I/II study were mainly patients with Hodgkin lymphoma 9, 10. Finally, we speculate that some chemotherapy‐refractory tumors may express higher levels of PD‐L1 and TIL markers, allowing them to be acceptable candidates for anti‐PD‐1/PD‐L1 immunotherapy.

A TME populated by suppressed/exhausted effector T‐cells is another component essential for a successful anti‐PD‐1/PD‐L1 immunotherapy approach 11, 17, 18, 19, 36. In our study, levels of the TIL markers CD3, CD8, CD45RO, and FoxP3 varied across indications, showing generally highest values in ganglioneuroblastoma and lowest values in nephroblastoma. Notably, during a case‐by‐case review, the specimens with highest TIL markers ‐except for nephroblastoma where biomarker expression was generally low‐ were not the same as those with the highest PD‐L1 levels. This information suggests that, in contrast to the high correlation of PD‐L1 and TIL observed in many adult tumor types 11, 36, PD‐L1 and TIL marker immunophenotypes in these pediatric tumors may not be governed by the same immunological mechanistic. To further confirm this concept, prevalence of the TIL marker FoxP3 could be used as an indirect assessment to check whether PD‐L1 drives a significant immunosuppressive role in the TME as it promotes T‐reg differentiation 38, 39. FoxP3 was the marker with the lowest levels across all six immune parameters, proposing that the scant amounts of FoxP3 should reflect –among other factors – a lack of PD‐L1 stimulation. Pigment interfered with the DIA performed in neuroblastoma and osteosarcoma, indicating that new disease‐specific algorithms were required. However, the low amount of FoxP3 visually evaluated (data not shown) suggested that the outcome using a disease‐specific algorithm will not be different from that already observed in the other four indications. To put in perspective how low the magnitude of our pediatric FoxP3 quantifications was, we have compared our pediatric values to those from an indication approved for anti‐PD‐1/PD‐L1 immunotherapy. The percentage of FoxP3 positive cells per all analyzed cells in neuroblastoma and ganglioneuroblastoma (indications with the highest numbers) was 0.4%, way below the 4.4% reported in a similar DIA in NSCLC 15. In both studies, FoxP3 was analyzed using the same clone (236A/E7), showing similar nuclear staining pattern and intensity when comparing FoxP3 scans from both studies (unpublished data). Therefore, it is unlikely that variations in their respective IHC protocols could explain the substantial reduction of FoxP3 detected in our pediatric study.

In conclusion, the low amounts of FoxP3 present in our pediatric samples in conjunction with the discrepancy between cases showing the highest levels of PD‐L1 and TIL markers suggested that PD‐L1 may not play a significant immunosuppressive role in our investigated pediatric tumors. These arguments speak against further development of anti‐PD‐1/PD‐L1 monotherapy in these indications. However, circumstances could be different if pre‐treatment could enhance the induction of PD‐L1 and TIL markers in tumors to allow subsequent anti‐PD‐1/PD‐L1 therapeutic intervention 6. Also, it is important to remember that the antitumor effect of immunotherapies is not only dependent on the quantity of the immune cells present in the tumor, but also on the quality and function of these cells, aspects not evaluated in this morphological study 6.

The only innate immune marker investigated in this study, CD68, showed variable levels across indications depending on the parameter used to express results. However, ganglioneuroblastoma and nephroblastoma were the indications with the highest and lowest median values using the two parameters, respectively. The percentage of positive cells per all analyzed cells of 5.1% in ganglioneuroblastoma was lower than the 8.6% detected in a similar DIA in NSCLC 15, and the relevance of these values to guiding anti‐PD‐1/PD‐L1 immunotherapy development remains unknown. Unfortunately, our procured tissue samples did not have additional clinical outcome information that could help us understand if CD68 has predictive or prognostic value. In addition, due to the nature of the investigated tumors, where identification of the TME was not feasible, TME compartmental quantification of CD68‐positive cells, which would help to understand the role of TAMs in these tumors, could not be performed 25.

Further phenotypic analyses using our dataset of individually segmented cells in registered whole sections could explore the spatial relationships of the analyzed cell populations beyond the global densities and percentages presented here, allowing us, for example, to assess tissue heterogeneity in order to identify niches of TIL and PD‐L1 enriched areas that could be relevant for PD‐1/PD‐L1 immunotherapy.

Analysis of clinical trial samples with PD‐1/PD‐L1 immunotherapy outcome information would have been the ideal set‐up for our study, but such a sample set was not available to us. For that reason, we conducted our proof‐of‐concept DIA study to assess the landscape of pediatric solid tumors for PD‐1/PD‐L1 immunotherapy analyzing high‐quality commercially available pediatric tissues from tumor resections acknowledging their limited clinical information, including lack of PD‐1/PD‐L1 therapeutic outcome.

In summary, considering the limitations of having analyzed small cohorts from these five solid tumor indications, the presented PD‐L1, TIL, and CD68 IHC data constitute the first quantitative screen using IHC DIA in pediatric oncology, proposing that the immunophenotype of the pediatric indications analyzed may not validate the PD‐L1 IHC biomarker patient selection strategy used for anti‐PD‐1/PD‐L1 monotherapies in adult cancer, while recommending focusing on other pediatric cancer groups.

## Author contributions statement

VD was project manager for tumor IHC and reviewed the manuscript. IK performed pathology review, manual annotations and manuscript review. SL was project manager for DIA, carried out data analysis and management, and reviewed the manuscript. MAS conceived and designed the study, interpreted data, and wrote and reviewed the manuscript. THT analysed data, performed statistical analysis, and wrote and reviewed the manuscript. NT developed the image analysis method, and wrote and reviewed the manuscript. MvK carried out the bridging IHC study. BvP carried out the bridging IHC study and reviewed the manuscript. JZ developed the image analysis method, and wrote and reviewed the manuscript.

## Supporting information


**Supplementary figure legends**

**Figure S1.** Boxplots showing percentages of biomarker positive cells/all analyzed cells per region of interest

**Figure S2.** Representative images of CD3, CD8, CD45RO, CD68, FoxP3, and PD‐L1 immunohistochemistry in ganglioneuroblastoma, neuroblastoma, osteosarcoma, rhabdomyosarcoma, and nephroblastoma

**Figure S3.** Correlation of PD‐L1 percentage positive cells and other immune markers in the centers of the tumors

**Figure S4.** Clustered heatmaps of the individual cases based on standardized expression levels of the individual marker percentages across investigated indications

**Table S1.** Percentage of PD‐L1 and FoxP3 positive cells in tumor compartments from Treated NSCLC
