# Supplementary material for: Biomarker recommendation for PD‐1/PD‐L1 immunotherapy development in pediatric cancer based on digital image analysis of PD‐L1 and immune cells
Source: J Pathol Clin Res. 2020 Jan 10;6(2):124–37. doi: 10.1002/cjp2.152 (PMC7164376; doi:10.1002/cjp2.152)
Supplement: Supplementary file 1 — Supplementary figure legends [file CJP2-6-124-s006.docx]

**Biomarker Recommendation for PD-1/PD-L1 Immunotherapy Development in Pediatric Cancer Based on Digital Image Analysis of PD-L1 and Immune Cells**

Silva MA *et al*, *J Pathol Clin Res*, DOI 10.1002/cjp2.152

**Supplementary Figure Legends**

**Figure S1.** Boxplots showing percentages of biomarker positive cells / all analyzed cells per ROI, which include positive and negative immune cells as well as tumor cells, for all investigated biomarkers across all investigated indications. Horizontal lines mark indication-pairs where the differences in percentage are statistically significant and corresponding adjusted p-values are labeled above.

**Figure S2.** Representative images (20x) of CD3, CD8, CD45RO, CD68, FoxP3, and PD-L1 immunohistochemistry in ganglioneuroblastoma, neuroblastoma, osteosarcoma, rhabdomyosarcoma, nephroblastoma and their corresponding image analysis segmentation overlays. For each indication, the same case and similar regions have been selected. The colors indicate blue for negative nuclei, brown for CD3, CD8, CD45RO, CD68, and FoxP3 positive cells; and yellow, orange, and red for PD-L1 positive cells of increasing stain intensity. Scale bar represents 50 μm.

**Figure S3.** Correlation of PD-L1 percentage positive cells and other immune markers in the centers of the tumors. The spearman correlation coefficients (SCCs) and their corresponding uncorrected *p*-values are given by SCC and p, respectively.

**Figure S4.** Clustered heatmaps of the individual cases (y-axis) based on standardized expression levels of the individual marker percentages across investigated indications**.** Red or blue indicates a higher or lower than average levels, respectively.
