# Supplementary figures and images for: Biomarker recommendation for PD‐1/PD‐L1 immunotherapy development in pediatric cancer based on digital image analysis of PD‐L1 and immune cells
Source: J Pathol Clin Res. 2020 Jan 10;6(2):124–37. doi: 10.1002/cjp2.152 (PMC7164376; doi:10.1002/cjp2.152)

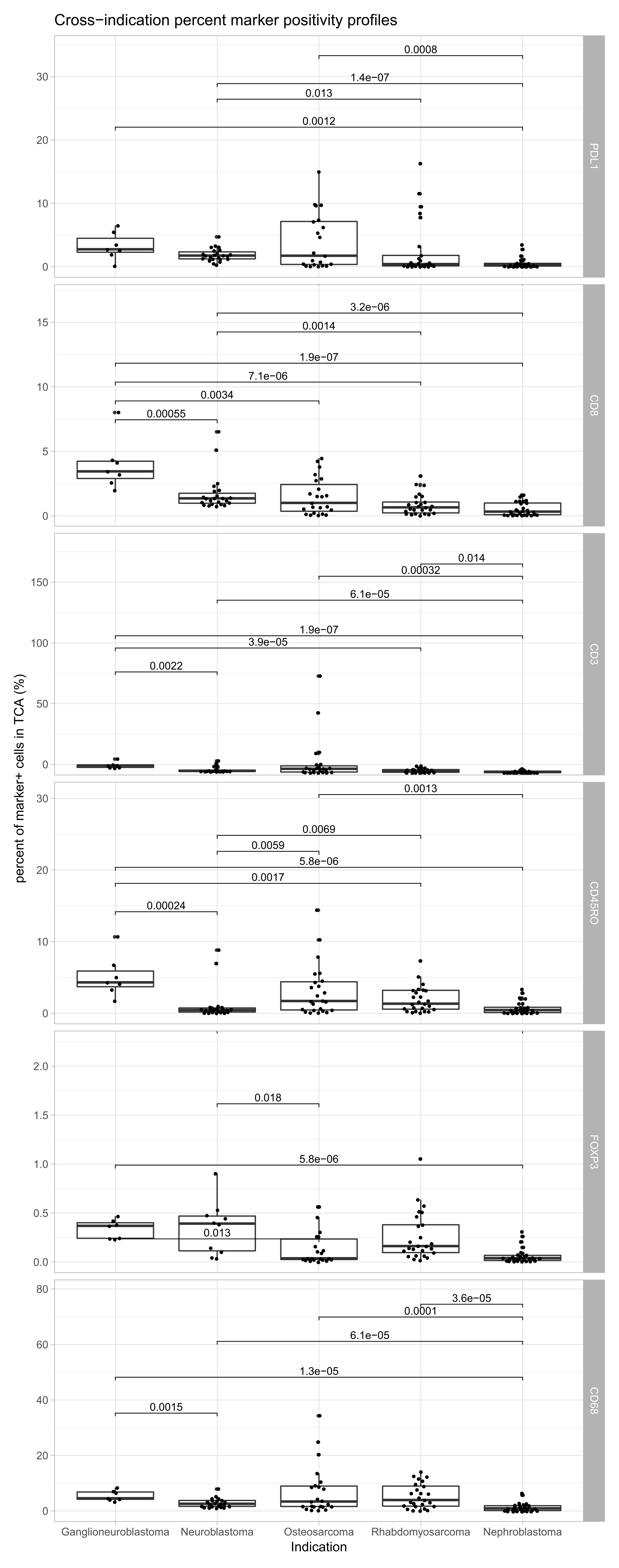

Supplement: Supplementary file 2 — Figure S1. Boxplots showing percentages of biomarker positive cells/all analyzed cells per region of interest [file CJP2-6-124-s001.tif]

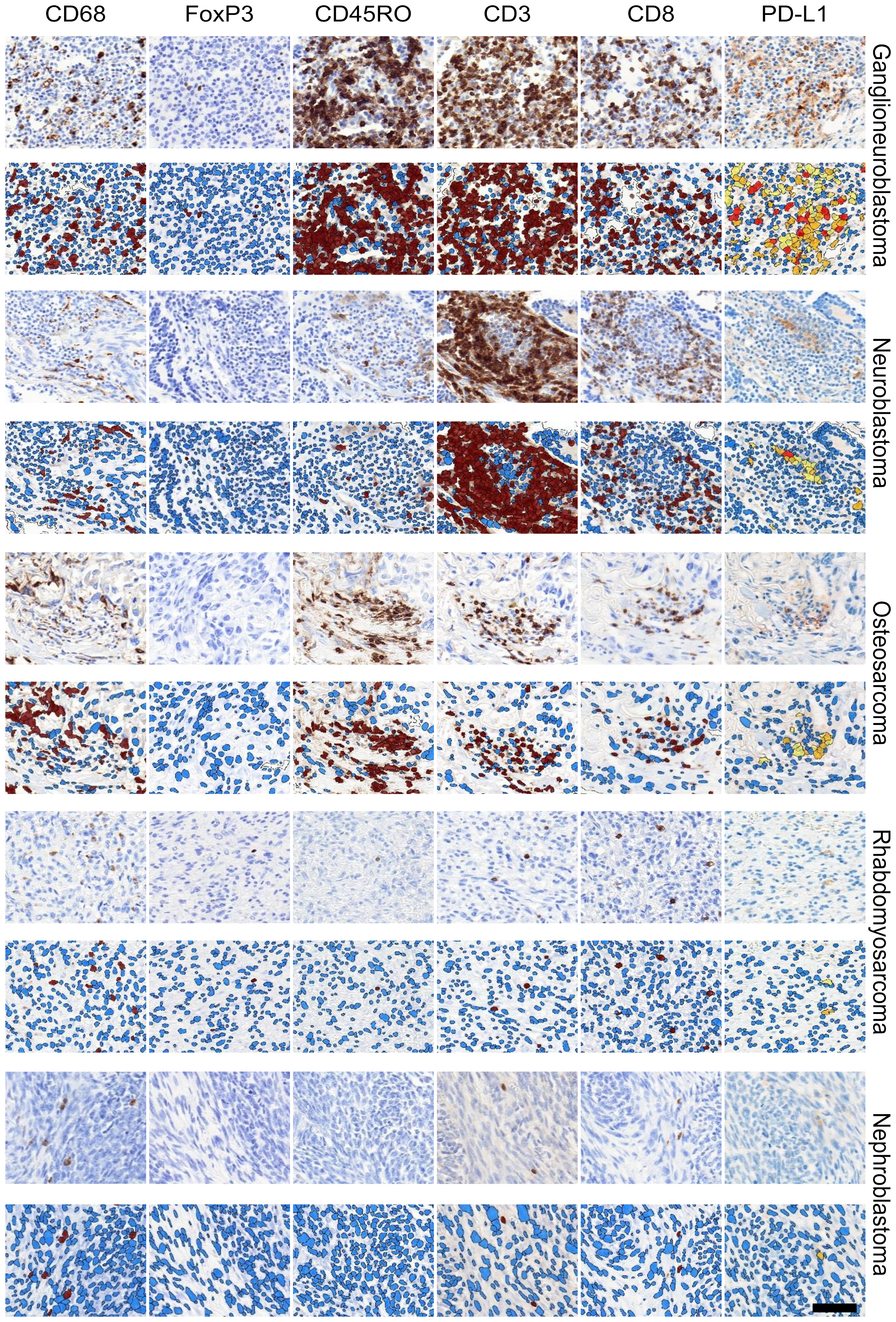

Supplement: Supplementary file 3 — Figure S2. Representative images of CD3, CD8, CD45RO, CD68, FoxP3, and PD‐L1 immunohistochemistry in ganglioneuroblastoma, neuroblastoma, osteosarcoma, rhabdomyosarcoma, and nephroblastoma [file CJP2-6-124-s002.tif]

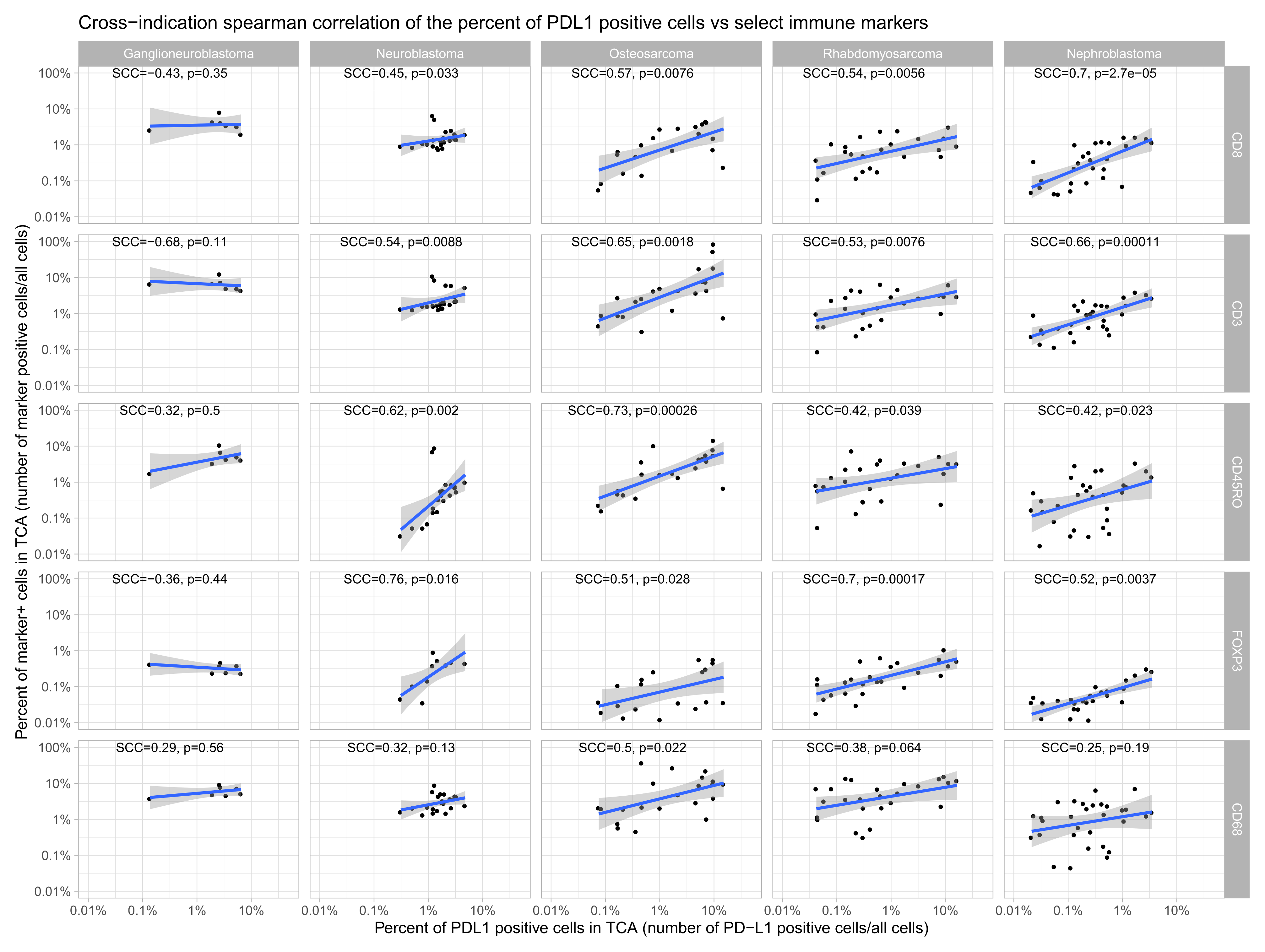

Supplement: Supplementary file 4 — Figure S3. Correlation of PD‐L1 percentage positive cells and other immune markers in the centers of the tumors [file CJP2-6-124-s003.tif]

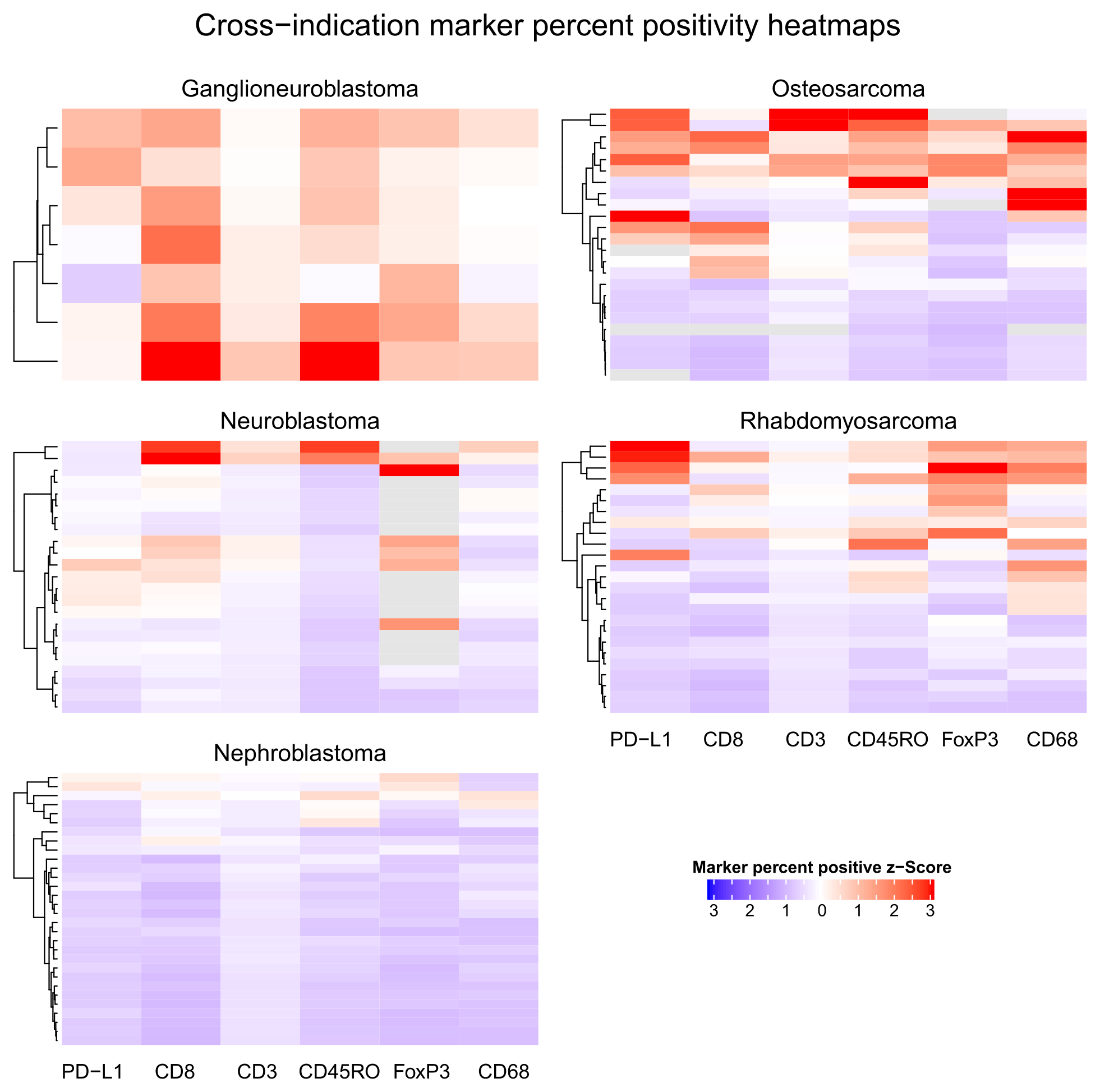

Supplement: Supplementary file 5 — Figure S4. Clustered heatmaps of the individual cases based on standardized expression levels of the individual marker percentages across investigated indications [file CJP2-6-124-s004.tif]
