# Supplementary material for: Biomarker recommendation for PD‐1/PD‐L1 immunotherapy development in pediatric cancer based on digital image analysis of PD‐L1 and immune cells
Source: J Pathol Clin Res. 2020 Jan 10;6(2):124–37. doi: 10.1002/cjp2.152 (PMC7164376; doi:10.1002/cjp2.152)
Supplement: Supplementary file 6 — Table S1. Percentage of PD‐L1 and FoxP3 positive cells in tumor compartments from Treated NSCLC [file CJP2-6-124-s005.docx]

**Biomarker recommendation for PD-1/PD-L1 immunotherapy development in pediatric cancer based on digital image analysis of PD-L1 and immune cells**

Silva MA *et al*. *J Pathol Clin Res* DOI: 10.1002/cjp2.152

**Table S1:** Percentage of PD-L1 and FoxP3 Positive Cells in Tumor Compartments from Treated NSCLC

|  | PD-L1 | | | | FoxP3 | | | |
| --- | --- | --- | --- | --- | --- | --- | --- | --- |
|  | Definiens DIA Results | | Flagship DIA Results | | Definiens DIA Results | | Flagship DIA Results | |
|  | TME | TCA | TME | TCA | TME | TCA | TME | TCA |
| Average ± SD | 34.9 ± 24.3 | 25.0 ± 33.8 | 27.9 ± 22.1 | 30.1 ± 35.7 | 3.6 ± 2.7 | 1.9 ± 1.2 | 2.5 ± 1.9 | 1.2 ± 0.9 |

Results from a non-small cell lung cancer (NSCLC) study performed in tissues from a second line therapy cohort (n=19). DIA: digital image analysis; TME: tumor microenvironment; TCA: tumor cell area
